# “*Eternally restarting*” or “*a branch line of continuity*”? Exploring consequences of external shocks on community health systems in Haiti

**DOI:** 10.7189/jogh.11.07004

**Published:** 2021-03-10

**Authors:** Pooja Sripad, Alain Casseus, Sarah Kennedy, Benito Isaac, Kenia Vissieres, Charlotte E Warren, Ralph Ternier

**Affiliations:** 1 Population Council, Washington DC, USA; 2 Zanmi Lasante, Mirebalais, Haiti

## Abstract

**Background:**

Community health systems (CHS) are integral in promoting well-being in humanitarian settings, like Haiti, a country plagued by disruptive socio-political and environmental shocks over the past two decades. Haiti’s community health workers (CHWs) as critical intermediaries have persisted throughout these contextual shocks. This study explores how shocks influence CHS functionality and resilience in Haiti.

**Methods:**

We applied an inductive and deductive qualitative approach to understand the lived experience of CHS actors. A desk review of peer-review and grey literature searched 393 and identified 25 relevant documents on community health policies, guidelines, and strategies implemented over the last fifteen years in Haiti. In-depth interviews with policy and program stakeholders (n = 12), CHWs (n = 24), and CHW supervisors and community health auxiliary nurses (n = 15) were conducted.

**Results:**

Various shocks – political transitions, natural disasters, and disease outbreaks – describe Haiti’s protracted complex humanitarian setting and reveal distinct influences on CHS functionality (challenges and enablers), resilience, and mediating factors (eg, policy, financing, governance, parallel systems). Consequences of civil unrest and lockdowns (political transitions), internal displacement and infrastructural damage (natural disasters), and livelihood depletion and food insecurity (natural disasters and disease outbreaks) affect CHS functioning. CHW resilience is rooted in their generalized scope of work, intrinsic motivation, history in the community, trusting relationships, self-regulatory capacity, and adaptability. Mental health and safety among CHS actors and communities they serve pose challenges to CHS functionality and resilience, while reinforcing collaborations that promote CHW coverage and support and sustain CHS. Participants recommended government support for CHWs, collaborations stewarded by the government and complemented by partners, sub-national autonomy, and integration of disaster preparedness for all CHWs.

**Conclusions:**

Political transitions, natural disasters, and disease outbreaks in Haiti continue to profoundly influence CHS functioning, despite mitigating policy and programming efforts. This study documents the relevance of CHS in maintaining primary health care for a country in protracted crises and suggests that propositions of CHW resilience can be explored in complex humanitarian settings globally.

Community health systems (CHS) are integral to promoting the well-being of populations in protracted humanitarian settings, such as Haiti, a country plagued by disruptive shocks including political transitions, natural disasters, and disease outbreaks over the past two decades. Increasingly, CHS reflect a “set of local actors, relationships, and processes engaged in producing, advocating for, and supporting health in communities” as an extension of the broader health system [1]. Community health workers (CHWs), critical actors within CHS globally, provide a range of reproductive, maternal, newborn and child health (RMNCH), and primary health care (PHC) including education, social support, and health promotion. As intermediaries between community members and facility-based clinical personnel, CHWs are defined as frontline workers with up to six months of initial training who provide care in community settings particularly for low-income or rural communities with limited access to facility-based care [2]. As community members themselves, CHWs understand the local context, including barriers and facilitators to health care, and can facilitate effective linkages to care, particularly in humanitarian settings [3].

While CHWs are increasingly recognized as essential to reducing service coverage gaps of under-resourced health systems and improving outcomes, there is limited understanding of their lived experience through crises [3-5]. In contexts where political instability and insecurity are normalized, health policy and program contexts comprise parallel governmental and non-governmental health systems that are influenced substantially by donors, international agencies, and/or military presence [6-8]. Natural disasters and disease outbreaks unveil health systems shortcomings and expose the vulnerabilities and risks faced by frontline health workers, including inadequate personal protective equipment, supervision, and mental health support [5,9]. Though shocks affect health systems, there is little empirical research on how and to what extent CHS and CHWs function under heightened levels of stress.

“Resilience”, a notion that operates at the population well-being, individual coping, and health and social systems levels, offers a useful lens to understanding CHWs experiences. Resilience is defined as a dynamic set of capacities that enable a person, household, community, or health system to withstand and recover from adversity [10-12]. At the individual level, characteristics of resilience include personality traits (eg, compassion, openness to uncertainty, adaptability), capacity to self-regulate and relate to others, social and structural supports, and the length of exposure to adversity [13,14]. Attributes of resilience of health systems include awareness, self-regulating capacity, integration of diverse actors and processes, and adaptiveness [15]. Contextualizing resilience in Haiti’s CHS and among CHWs requires in-depth exploration and historical review of how these actors perceived and lived shocks.

## Community health in Haiti

Community health in Haiti is the primary source of health counseling and services for much of the population, with an intended one CHW per 1000 rural and 2000 urban population, respectively. CHWs’ roles have shifted over time from disease-specific “accompagnateurs,” to basic “Agent de Santé” (health worker) care for infant, child, and maternal health issues, community-wide immunizations, and community education [16], to the current generalized cadre known as “agents de santé communautaire polyvalent” (ASCPs). ASCPs counsel, provide health services, and refer (and in some cases accompany) individuals to health facilities for a variety of health issues, including nutrition, RMNCH, communicable and non-communicable diseases, mental health, and other emergencies. ASCPs are expected to make 100 home visits per month during which they provide health education, probe individuals about their family’s overall health, answer questions, document/report conditions, offer direct services, and refer or follow up on counter-referrals. They connect with individuals over the phone and through community meetings, rally posts, and school-based health programming.

Haiti’s administrative and environmental instability is exemplified in a series of shocks coincident with fluctuations in health sector priorities and financing [17]. This historic and current volatility has led Haiti’s health care system to evolve as a heterogeneous (“parallel”) system with the lowest regional average health indicators [18]. Little is known about how these political and external factors affect community health policy, programming, and performance. The continued efforts of CHS actors suggest a level of resilience that merits investigation. The purpose of this study is to explore how political and environmental shocks influence CHW policy and program sustainability in Haiti. Specifically, we sought to understand (a) the types of shocks experienced in Haiti; (b) how these shocks have affected the functionality of CHS and CHWs, (c) what constitutes resilience among CHWs and CHS and (d) what recommendations can support CHW performance and CHS in preparation for future shocks.

## METHODS

We conducted a mixed inductive and deductive qualitative study, drawing on a desk review of the extant literature and in-depth interviews (IDIs) to analyze the lived experiences of CHS actors through various shocks and offer recommendations to further strengthen the resilience of CHWs in Haiti and other humanitarian settings.

This study is nested within the Frontline Health project’s research portfolio, which focuses on operational and contextual considerations that affect the institutionalization of community health. Study sites were selected in collaboration with Zanmi Lasante and government stakeholders at the Ministère de la Santé Publique et de la Population (MSPP) to offer insights into unique humanitarian challenges to CHS in Haiti. We collected information at the national, sub-national/departmental levels (the Artibonite and Centre departments), and local CHW programmatic levels.

### Data sources

The desk review aimed at describing different CHW approaches in Haiti over the last 10-15 years, historical challenges through varied shocks, and insights into sustainability implications of parallel systems. Peer reviewed articles, grey literature, and Haiti national health policy documents that were published between 1 January 2004 and 31 December 2019 were considered for inclusion. Eligibility was restricted to English-language open access articles and French-language documents including policies, guidelines, or reports. Search criteria on PubMed and Google Scholar included “Haiti”, “community health worker”, “health policy”, and “health system”; additional documents were extracted using a snowball approach through referenced pieces and expert consultation in Haiti. In total, 25 peer-reviewed articles, 19 grey literature pieces, and six national health policy documents were identified.

In-depth interviews (IDIs) were conducted with ASCPs of varied experience (n = 24), ASCP supervisors including community health and auxiliary nurses in two departments (n = 15), and current or recent policy stakeholders at the national and department levels active over the past five years (n = 12). IDIs explored each group’s lived experience through various policy and programming phases and shocks identified from the desk review. Interviews reflected the challenges experienced by and resilience of CHS (primarily over the last 10 years), probing on the quality and nature of CHW-community relationships in these shifting circumstances. Interviewers also elicited recommendations on aspects of support.

Participants were recruited by community gatekeepers at Zanmi Lasante and most were interviewed in-person in a community or private workplace setting. Two national policy stakeholder interviews were conducted by phone given the civil unrest in late 2019. Research assistants (RAs) with social sciences backgrounds and a four-day training on ethics and study protocols conducted the informed consent process with potential participants, explaining the voluntary nature of the study, the risks and benefits, confidentiality procedures, and their ability to withdraw at any time. After obtaining written consent, RAs carried out the interview in the language of the participant’s choice (French, Kreyol, or a mix). Interviews were audio-recorded digitally, transcribed, and translated into English. Ethics approval for this study was provided by the Institutional Review Board in the Population Council, New York, USA (*p879*), and Zanmi Lasante, Mirebalais, Haiti (*ZLIRB2732019*).

### Analysis

A mixed inductive and deductive approach was applied to analyze and triangulate our data. Desk review documents were summarized in memos, which were then coded alongside interview transcripts using NVivo 12 software. A codebook was developed by co-authors after reading a subset of transcripts and highlighting emergent themes. Guided by study questions and transcripts, the following elements were included: contextual and environmental shocks that affected CHS (themes: political transitions, natural disasters, disease outbreaks), CHS functionality (themes: structure and governance, challenges and enablers of CHWs), and notions of resilience (themes: value of CHWs, trust and relationships between communities and CHWs). Following an iterative process of reading and categorizing, a final codebook was applied to all the data by two coders. Corroborating findings describing each thematic area across respondent perspectives as well as linking to documentation of events and community health policy and strategies allowed for development of a mid-level theory and select propositions related to shocks-to-resilience pathways. Consensus suggestions are presented as recommendations to sustain CHS functionality in humanitarian settings.

## RESULTS

### Community health systems shocks and policy context

**Figure 1:**
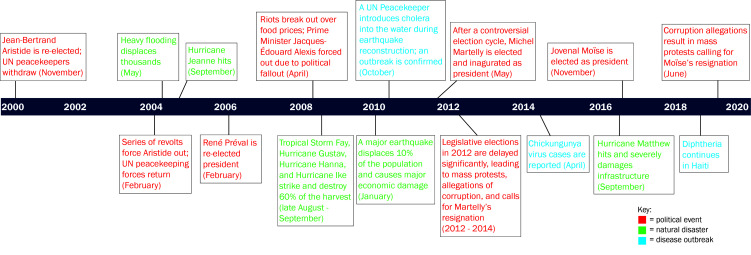
Timeline of political transitions, natural disasters, and disease outbreaks in Haiti from 2000-2020.

Three broad types of shocks emerged from the documents review (n = 50) and interviews (n = 51): political transitions, natural disasters, and disease outbreaks. The desk review relates the types of shocks to aspects of the CHS structure and organization including: the parallel government-led and non-governmental organization (NGO)-supported health systems (n = 12), training and support for CHWs in their roles and health areas (n = 14), and shock-specific governance and accountability (n = 12). Patterns in **Figure 1** reveal a periodicity in political transitions, natural disasters, and disease outbreaks that describe the protracted humanitarian setting.

These events follow historical trends of leadership turnover and uprisings, intertwined with donor influence that democratized Haiti in the 1990s, and persist through socioeconomic fluctuations and sustained poverty in the 21st century [19-22]. Alongside shocks, donor financing cycles and repeated “restarting” of health policy and guideline development (**Table 1**) influenced the functioning of the CHS.

**Table 1:** Key Haitian health-related policy documents

Policy/strategy/guideline	Developed or supported by	Objective/Intention	Year
Strategic Plan for Health Sector Reform (2003-2008)	MSPP	This document details the country's initial health-policy framework broad goals for health care provision and health sector reform, including guaranteeing the population’s access to essential drugs, developing human resources for health, and decentralization of the health system in Haiti.	2004
Master Plan for Health/Plan Directeur de Santé 2012-2022	MSPP	This 10-year plan for health care service provision and delivery outlines broad goals such as improving and extending essential health care, infrastructure, and human resources. Strengthening PHC networks within the country is a key component. The community health structure and duties of ASCPs are also outlined.	2012
National Health Policy/La Politique National de Santé	MSPP	This policy outlines MSPP's plans to develop a health system capable of providing full health coverage that can meet the basic health needs of the population while promoting the use of modern and traditional medicines. Two specific goals of the policy were to increase the budget allocated to health and streamline use of available resources by aligning donors with national priorities.	2012
Model Referral Networks	MSPP/USAID	The MSPP and USAID’s Maternal and Child Survival Program collaborated on the project, Services de Santé de Qualité pour Haiti (SSQH) project to develop a referral and counter-referral systems and protocols. These tools were piloted through the project, adopted and scaled up at the national level.	Project duration: 2013-2016
Essential Services Package Manual (ESP)/ Paquet Essential de Services et Soins de Santé (PES)	MSPP/USAID	This more recent iteration is an update from the ESP detailed in the 2004 Strategic Plan, developed with USAID, that outlines all essential health services the population should receive, and how to consolidate and improve existing services.	2015

MSPP – Ministère de la Santé Publique et de la Population

“A lot of policy writing… there is something blocking us… the structures that need to be set up to allow the writing to come true.” *(Department program stakeholder)*

“It goes back to remaining on course with the [MSPP’s] strategy….The Ministry, from 2016, has advocated for establishing Family-centered Health Teams…The funds provided by partners are of great help, but believing in the overall strategy is equally helpful.” *(National policymaker)*

“We do not do continuity well. We are eternally restarting, restarting is the worst thing that we could do to the population. In Haiti, transitions are never about continuity, it’s all about doing in what’s in my best interest.” *(Department policy stakeholder)*

While community health policy implementation constraints exist in non-shock periods, there was cross-perspective concurrence on the importance of CHWs and CHS in crisis periods.

“In times of humanitarian crisis, health workers are very helpful to the local communities. They do the footwork, function as “arms” of the institutions…on the field, they allow the institution to have a better understanding of the problems in affected areas. They relay valuable information back allowing the institution to respond faster to epidemic cases or health related problems.” *(National policy maker)*

### Consequences by shock type

**Figure 2:**
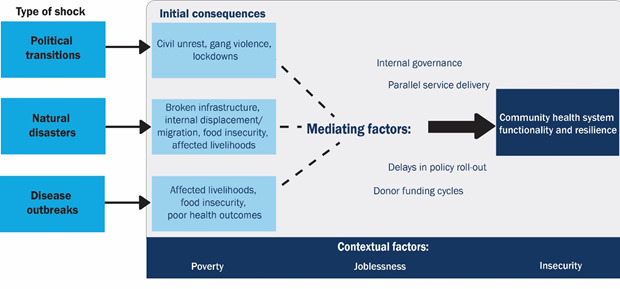
Shocks, consequences, and mediating factors affecting Haiti’s community health system functionality and resilience.

Political transitions, natural disasters, and disease outbreaks in Haiti reveal distinct downstream consequences that affect CHS functionality (challenges and enablers), and resilience (**Figure 2**). All respondents situated these pathways in an underlying context of poverty, insecurity (intermittent gang violence), and joblessness.

#### Political transitions

Political transitions over the last 10 years in Haiti described by ASCPs, ASCP supervisors, and policy stakeholders are typically followed by periods of civil unrest, ranging from peaceful stand-ins to violent riots across the country. During these times, gang activity –including barricades and roadblocks– and government-initiated lockdowns are commonplace. Administrative shifts lead to months of delay in community health policy decisions, funding, and program implementation and support. These shifts often result in highly skilled health workers leaving the country, and constrain the ability of parallel and shifting NGO-managed health care to sustain community-based PHC. In some cases, political transitions are accompanied by shifts in donor funding and NGO presence, affecting CHW salary and support.

“When this [political transitions] happens, it affects not only the cities but also the rural communities and they impact the activities. For example, you have a rally post and you have everything -coolers, vaccines- and you show up at the station…and the mothers are afraid to come due to the tension in the streets.” *(ASCP supervisor)*

“Say an NGO decides to leave, the workers don’t know who’s going to replace it… This causes problems for everybody…. the treaty hasn't been signed yet or it may need to be brought to the attention of the new Minister...this causes delays.” *(Department level policymaker)*

CHS functionality faces a range of challenges including suspension of resource transfers (equipment, medicines, and supplies) and payment distribution. These factors resonate with the experiences of limited instrumental support and protection described by CHWs continuing to work for pay or free. Referral challenges include the breakdown of health care provider networks that exist in Haiti’s parallel service delivery system, which restricts patient access to necessary health care (eg, pregnancy, childbirth and other routine care).

“There were areas completely cut off; everywhere was blocked... For one month we could not get to “Zone Tenet” for a vaccination activity because the [Department] direction could not open its doors; the vaccines could not arrive from Port-au-Prince...This paralyzed our activities. We all felt it.” *(ASCP)*

“Transportation fare increases when you have political unrest…, You go out and they tell you it’s 75 gourdes, when you know it was 50 gourdes. If the county was stable, it would be 5, 10 gourdes, now the drivers increase the fares as they go.” *(ASCP)*

“We get support from our partners or donors, not the government…when you have a transition that lasts a while, it affects community health because most personnel are placed on stand-by and some volunteer to work [as if ] under normal conditions…[In 2012 transition]…there were issues putting a government in place and [health care service delivery] networks where the partners’ mandates had expired… communes that had networks became dysfunctional and it took a long time…for our partners to obtain the authorizations needed.” *(National policymaker)*

The restricted mobility of CHWs relate to fears for one’s own life, particularly when kidnappings for ransom occur. In addition to the infrastructural and gang-mediated roadblocks and public violence, this fear renders some CHWs unable to work at all. In some situations, CHWs describe continuing to be there for their communities through phone calls and decision support for referrals and accompaniment to life-saving care.

“Due to the political turmoil, sometimes I felt uncomfortable about ASCPs and I going to work. I made a lot of sacrifices to come – go through the woods to avoid the roads, almost getting hit by rock. Sometimes it didn't impact me because it was my job to serve people in the community and they were really traumatized when I went to talk to them.*” (ASCP supervisor)*

#### Natural disasters

The second shock type, natural disasters, particularly focused on Hurricanes Jeanne and Matthew (2004 and 2016) and the 2010 earthquake. These shocks lead to periods of internal displacement and migration, depleted infrastructure, job loss, and food insecurity and affected livelihoods (eg, crops destroyed). The shocks were mitigated by increased donor funding and external support to the national government that manifested in varied collaboration in CHS at different levels. While collaboration of international agencies often facilitated access to supplies/equipment and temporary housing, and short-term military presence focused on tertiary care provision, international support for CHS functionality produced mixed results [23,24]. For example, when coordination was not communicated clearly and donor-supported, CHWs moved into highly affected areas and sometimes experienced hostility from local authorities and health care providers.

“Generally, when we [MSPP]respond to disaster, we set up cells in concert with local officials and civil protection workers in the affected area and work on ways to find solutions to the various problems.” *(National policymaker)*

“There are those that are always in the mix. Once you hear disaster, they are always there…setting up meetings, evaluations. These NGOs, donors are always at the forefront.” *(Department policy maker)*

“You may get somewhere where is much devastation and they [existing healthcare providers] wonder why you are there when there are already hospital staff present. Or they ask [skeptically], what are you going to do for them, you know, I always try to keep an open mind.” *(ASCP)*

Some CHW routine activities halt in response to infrastructural challenges that inhibit movement and door-to-door services, while other activities, including phone-based communication and infection prevention and hygiene advice, increase. While CHWs are not always given refresher training nor adequate supplies, participants concur that there is a recognition that CHWs continue functioning as the main sources of health care in crises.

“The health workers’ job is to educate, to mobilize the local community. If there’s a natural disaster, a CHW might have to work under more pressure but the workload has not increased. Given the forecasted hurricane or earthquake, s/he will go to door-to-door to inform the locals on hygienic measures, on precautions to take … Sometimes we send CHWs out without the proper materials - this is the biggest difficulty…after a hurricane, when CHWs arrive the people to ask them for water treatment solutions and other items which they do not have and people may turn the CHWs away…” *(Departmental policymaker)*

“It [natural disaster] gets people moving, everybody has to own up to their responsibilities, and see how they can lend a hand, encourage the health workers to give more of themselves and help those in need, those that need assistance...” *(ASCP supervisor)*

“If it [river] overflows [after a natural disaster], you cannot cross it. If we needed to get a pregnant woman across… whether or not she is dying, we must wait till the water level recedes to be able to take her to the hospital.” *(ASCP)*

Psychological shock and trauma was raised as a critical challenge following natural disasters among all CHS actors interviewed. They spoke of their own mental well-being and ability to function as related to the simultaneous feelings of helplessness around their capabilities to help their communities. In part, mental stress experienced by CHWs and communities alike is related to the death of someone close, displacement, unemployment, and lack of access to food [25]. Perspectives of CHWs and their supervisors also reveal an acceptance of adversity that helps them to continue doing what they can by emphasizing preparedness and offering empathy and social support to their communities.

“Psychologically I was not okay [after Hurricane Mathew]. I think it's the same for the CHWs as well, s/he is a person just like everyone else. It’s true that it is they are there to help despite any circumstances, but I think this will affect a health worker psychologically, socially, economically and in terms of sanity.” *(National policymaker)*

“When you have disasters like this [natural disasters], there are people who live in at-risk areas, things usually go from bad to worse for them; they tend to lose everything. When they bring their grievances to you…you cannot repair their houses, you cannot help them recover the things they lost. The only thing you can do is commiserate with them.” *(ASCP supervisor)*

“The locals have accepted the fact they cannot stop the hurricane from inflicting damages. They can only protect their livestock and we’ve show them how important it is to protect their personal documents by securing them in plastic cases and bags.” *(ASCP supervisor)*

#### Natural disasters

The second shock type, natural disasters, particularly focused on Hurricanes Jeanne and Matthew (2004 and 2016) and the 2010 earthquake. These shocks lead to periods of internal displacement and migration, depleted infrastructure, job loss, and food insecurity and affected livelihoods (e.g. crops destroyed). The shocks were mitigated by increased donor funding and external support to the national government that manifested in varied collaboration in CHS at different levels. While collaboration of international agencies often facilitated access to supplies/equipment and temporary housing, and short-term military presence focused on tertiary care provision, international support for CHS functionality produced mixed results [23,24]. For example, when coordination was not communicated clearly and donor-supported, CHWs moved into highly affected areas and sometimes experienced hostility from local authorities and healthcare providers.

“Generally, when we [MSPP]respond to disaster, we set up cells in concert with local officials and civil protection workers in the affected area and work on ways to find solutions to the various problems.” (National policymaker)

“There are those that are always in the mix. Once you hear disaster, they are always there…setting up meetings, evaluations. These NGOs, donors are always at the forefront.” (Department policy maker)

“You may get somewhere where is much devastation and they [existing healthcare providers] wonder why you are there when there are already hospital staff present. Or they ask [skeptically], what are you going to do for them, you know, I always try to keep an open mind.” (ASCP)

Some CHW routine activities halt in response to infrastructural challenges that inhibit movement and door-to-door services, while other activities, including phone-based communication and infection prevention and hygiene advice, increase. While CHWs are not always given refresher training nor adequate supplies, participants concur that there is a recognition that CHWs continue functioning as the main sources of health care in crises.

“The health workers’ job is to educate, to mobilize the local community. If there’s a natural disaster, a CHW might have to work under more pressure but the workload has not increased. Given the forecasted hurricane or earthquake, s/he will go to door-to-door to inform the locals on hygienic measures, on precautions to take … Sometimes we send CHWs out without the proper materials - this is the biggest difficulty…after a hurricane, when CHWs arrive the people to ask them for water treatment solutions and other items which they do not have and people may turn the CHWs away…” *(Departmental policymaker)*

“It [natural disaster] gets people moving, everybody has to own up to their responsibilities, and see how they can lend a hand, encourage the health workers to give more of themselves and help those in need, those that need assistance...” *(ASCP supervisor)*

“If it [river] overflows [after a natural disaster], you cannot cross it. If we needed to get a pregnant woman across… whether or not she is dying, we must wait till the water level recedes to be able to take her to the hospital.” *(ASCP)*

Psychological shock and trauma was raised as a critical challenge following natural disasters among all CHS actors interviewed. They spoke of their own mental wellbeing and ability to function as related to the simultaneous feelings of helplessness around their capabilities to help their communities. In part, mental stress experienced by CHWs and communities alike is related to the death of someone close, displacement, unemployment, and lack of access to food [25]. Perspectives of CHWs and their supervisors also reveal an acceptance of adversity that helps them to continue doing what they can by emphasizing preparedness and offering empathy and social support to their communities.

“Psychologically I was not okay [after Hurricane Mathew]. I think it's the same for the CHWs as well, s/he is a person just like everyone else. It’s true that it is they are there to help despite any circumstances, but I think this will affect a health worker psychologically, socially, economically and in terms of sanity.” *(National policymaker)*

“When you have disasters like this [natural disasters], there are people who live in at-risk areas, things usually go from bad to worse for them; they tend to lose everything. When they bring their grievances to you…you cannot repair their houses, you cannot help them recover the things they lost. The only thing you can do is commiserate with them.” *(ASCP supervisor)*

“The locals have accepted the fact they cannot stop the hurricane from inflicting damages. They can only protect their livestock and we’ve show them how important it is to protect their personal documents by securing them in plastic cases and bags.” *(ASCP supervisor)*

#### Disease outbreaks

The third shock type, disease outbreaks, draws on the recent experiences of cholera, chikungunya, diphtheria, and malaria (endemic). Though consequences and mitigating factors affecting CHS closely align with those of natural disasters, some are unique given outbreaks fall directly within a CHW’s scope of work. While the MSPP’s coordination of elevated donor funding and external and local implementing partner support for certain outbreaks (eg, cholera) appeared moderately functional given the influx of implementing partner projects that brought infection prevention supplies and support for WASH activities, other outbreaks (eg, diphtheria) lack similar investments (eg, vaccines, medicines) by the government.

“We fought it well…we hardly see cholera anymore….Every day we preached: how to behave, how to wash hands, how to drink treated water, how to keep hygiene measures and not defecate on the ground.” *(ASCP)*

“Our workload increased greatly, we used to go decontaminate homes. We had found support from other organizations that had been providing people of the community with aquatabs, chlorine, and spraying homes. The MOH coordinates efforts to educate and advise them the people.” *(ASCP)*

“Because of the [cholera] the CHW is forced to increase the number of his visits and takes much more time with people in the community. He is obliged to go and see the disabled, the elderly, and children…. All this causes him to spend a lot more time in the field.” *(National policy maker)*

Though CHWs typically function well as responders in disease outbreak situations, quickly mobilizing to educate, prevent, and treat (or refer) for the various illnesses, they face challenges in the community. Lost livelihoods or food insecurity are challenges to disease prevention and treatment. Few CHWs express concerns with routine activities taking a backseat and a fear that they may bring home an illness and infect their relatives. The lack of support in the form of additional payment or recognition for the risks taken by CHWs were described by few participants.

“During an epidemic, people are too busy worrying about other things to listen to you, to take part in the activities, because they are ill or their gardens [crops] are ruined, the water has carried away their livestock, they are quite disconnected from everything.” *(ASCP supervisor)*

### Resilience

Irrespective of the external shocks, CHWs and others in the CHS consistently exhibit resilience as integral actors in the sustaining PHC services in Haiti. Not only do they share an altruism, love for their work, and longstanding history within the community; these groups are intrinsically motivated and committed to community health. Most CHWs report working despite the insufficient standard monthly stipend of 12 000 HTG (100 USD). Consistently, CHWs self-initiated or were encouraged by community members to apply at various stages of community health program revitalization (from the disease specific sectors to the accompagneteur to the ASCP). Beyond the time lived in the community, CHWs previously engaged in related work such as teaching, working with other service organizations, or participating in church activities. Supervisors consider themselves as extensions of CHWs; some describe their commitment increased after witnessing and living through multiple shocks. CHWs maintain strong positive trusting relationships with patients and see themselves as bridges and leaders within the health system.

“When you are in this position, you are looked upon as a leader. You have a duty to lead..you develop close relationships with people, you are a counselor and a confidante...they trust you as a friend…It comes down to the relationships you build over time.” *(ASCP)*

“You have to know how to approach them, how to talk to them in the right way, tell them that you need them to live, and finally, they accept to sit with you…I have a close relationship with them [community]…I went three years without being paid…Some people wonder why I chose to stay; the reason is I want these patients to remain alive. They are my people.” *(ASCP)*

“If I didn't appear the patient would have died. I had a serum (ORS) in my purse, I gave it fast…[symptoms persist]…I discovered it was cholera. I told the people to hurry and take the patient to the hospital where they found care. I was so proud to have provided such a service that day.” *(ASCP)*

In the context of any shock, CHWs’ fears and constraints affecting their work lead to innovative solutions; all participants agree that CHWs still reach and interact with communities. They continue door-to-door visits as possible, use phone-based and digital communication, and inspire communities to “lend a hand.” They also mitigate travel risks for communities by working with local information sharing networks to refer patients to facilities through unblocked roads/routes to hospitals/clinics.

### Recommendations for sustainability

When asked how to support CHS (and CHW) functionality and preparation for future shocks, triangulated perspectives emphasize increased focus on community health as priority, departmental autonomy in decision-making, CHW involvement in identifying gaps and solutions, and increased CHW-support during/immediately following shocks. Prioritization of community health by the government, including line-item budgets, were seen as essential given the parallel systems of NGO and government service delivery and donor funding cycles (to ensure continued care after donors or implementing partners leave). Departmental governance structures should maintain some autonomy to finding localized solutions during crises when there are lags in distribution from central resource pools.

“The government should be strengthened because I can tell you that if it were not for the “NGOs” I do not know what the country would be like. If an “NGO” leaves, the programs have also died. So, I wish the government would support the programs when the “NGO” goes to ensure continuity.” *(ASCP)*

“Normally, CHWs have a job to do in promotion and intervention…immediately after a natural disaster, you have a high risk of infection and other outbreaks, they need supplies, and a little coaching, to be able to inform the locals on precautionary measures in order to avoid the worst.” *(Departmental policy maker)*

“The salary hasn’t changed; it has remained the same. The thing has changed is that we are getting more and more patients and they are asking more and more from us. With a new year coming in, ... Hopefully the government will look into the matter… and do something to improve our lives.” *(ASCP)*

Given CHWs possess intimate knowledge of the practical weaknesses and strengths of service provision, involving them in decisions can strengthen community health policy. For example, they articulate the need to elevate – within disaster preparedness mechanisms – the food insecurity concerns paramount during natural disasters and disease outbreaks. Recommendations to support CHWs generally include a livable salary that precludes them from needing a second job and affords them regular training, supported referral and counter-referral tools, protections, career growth and benefit opportunities, and continuous telephone-based and monthly supervisor meetings. In times of CHS shocks, CHWs require additional financial support for risks and unexpected costs, transport subsidies, and avenues to reach supervisors efficiently and supportive departmental oversight.

## DISCUSSION

This study developed a data-driven theory of how external shocks interact with policy and health systems environments to affect the functioning and resilience of CHS in Haiti. The cross-perspective lived experiences of shock types unveil similar yet distinct consequences affecting CHS functionality and resilience, including mediating factors – policy, financing, governance, and parallel systems. Insecurity, mobility restrictions, and constrained supply chains affecting CHWs ability to work emerge across all shock types. Civil unrest and lockdowns present unique challenges to CHWs and CHS during political transitions, while livelihood depletion and food insecurity comprise bottlenecks following natural disasters and disease outbreaks. Internal displacement and widescale infrastructural damage are critical in natural disaster contexts. Following shocks, mental health and safety among CHS actors and the communities they serve pose challenges to CHS functionality and resilience, while reinforcing collaborations that promote CHW coverage and support and sustain CHS. Amidst shocks, CHWs demonstrate resilience in their commitment to providing essential services to their communities.

CHS and CHWs were prevented from carrying out their routine functions during political instability compared to other shocks. This relates in part to their intrinsic role in natural disaster and disease outbreak scenarios, on the one hand, and adjacent role in situations of civil unrest and unpredictable violence, on the other. This barrier also relates to the breakdown of supply chains in often already weakened systems [26]. Despite the normalized insecurity in Haiti, where CHWs understand and adapt rapidly to circumstance, the scale of riots, roadblocks, lockdowns, kidnappings, violence, and militarized response that follow shifts in political power pose challenges to program implementation. In Haiti, the overlay of shocks destabilizes the contextual factors and undermines the CHS to continue supporting CHWs in their work [27]. Our findings resonate with data from other protracted complex humanitarian settings, where overlapping effects of conflict-driven insecurity across shock types often inhibit access to basic PHC including RMNCH services given the high risks faced by health workers [6].

In Haiti, resilience of CHWs and other CHS actors is rooted in their generalized scope of work, accepted value as longstanding trusted intermediaries, responsiveness to their communities, self-regulatory capacity, and adaptability. The variety of health topics and service areas covered and mechanisms through which CHWs engage with communities enable them to swiftly pivot focus of health educational and counseling sessions as needed. Lived experiences of CHWs in Haiti suggest adaptability as a critical enabler; CHWs tailor messages around infection prevention, nutrition, WASH, surveillance, and mental health following hurricanes, the 2010 earthquake, and outbreaks like cholera. Contrastingly, during these same shocks, challenges in performing routine activities with limited structural support and their own individual psychological processing of the shock affect CHW self-regulatory capacity. Similar enablers and challenges are observed in CHW involvement in preventing the spread of Ebola in West Africa, cholera in Yemen, and armed conflict settings of DRC and Afghanistan [7,28,29]. Embedding disaster preparedness in Haiti’s CHW training curricula offers a step to formally building CHS actor capacity to adapt to shocks. A CHW’s deep sense of belonging to a community, caring nature, duty, and presence in crises accrue over time [30]. This is exemplified in CHWs facilitating mental health care for communities battling various degrees of trauma following serial disasters in Haiti [31].

Mitigating governance and support factors affecting the resilience of the CHS in Haiti manifest in the degree to which CHWs cope with and manage effects of shocks for themselves and the communities they serve (self-regulatary capacity). Where there is adequate coverage and consistent support for CHWs, they are better equipped to readily respond to shocks. Despite existing community health policy implementation gaps in Haiti during shock and non-shock periods, collaboration within parallel health service delivery systems between sectors and partners (eg, government, NGOs, international agencies, and donors) is critical to ensure sustained support and coverage. Our findings around positive engagement in natural disaster and disease outbreaks suggest that complementary collaborations, comprised of government stewardship, community partnership, and complementary NGOs, historically enable short- and long-term CHS functionality and resilience in Haiti [32]. Irrespective of shock type, our data suggests a need to protect and support CHWs and their supervisors – financially, mentally, and materially – to promote CHS resilience. Future studies and community health policy and programming ought to consider all these aspects of support.

There are limitations to our study. The historical framing by self-report introduces potential recall bias of events that occurred years ago, yet the balance of current and forward-looking questioning reveal salient learning from prior shock experiences. We sampled CHWs in two out of 10 departments that received support from the government and NGO sectors, which may reflect a more resilient population compared to other CHWs. The policy/program stakeholder and supervisor interviews helped triangulate these perspectives alongside the desk review. Given Haiti’s unique situation of being geographically isolated, with high rates of poverty, low employment, and a normalized state of insecurity, transferability of our findings may be limited.

## CONCLUSIONS

Our propositions on how political transitions, natural disasters, and disease outbreaks affect the lived experience of CHWs and others in the CHS have implications for sustaining community relationships, routine services, and shock-related care (eg, infection prevention, mental health). This study reinforces the relevance of CHS in maintaining PHC for a country in protracted crises and describes CHW resilience resulting from their generalized scope, trusting nature, responsiveness, self-regulatory capacity, and adaptability. This mid-level theory can be further explored in similar complex humanitarian settings globally and can contextualize implementation research around CHS-supportive interventions in Haiti. As one policy maker states, “*Community health service a branch line, it is continuity.*”
